# Epileptic seizure prediction using successive variational mode decomposition and transformers deep learning network

**DOI:** 10.3389/fnins.2022.982541

**Published:** 2022-09-26

**Authors:** Xiao Wu, Tinglin Zhang, Limei Zhang, Lishan Qiao

**Affiliations:** ^1^School of Mathematics Science, Liaocheng University, Liaocheng, China; ^2^School of Computer Science and Technology, Shandong Jianzhu University, Jinan, China

**Keywords:** seizure prediction, successive variational mode decomposition, multiscale time-frequency analysis, BERT, intracranial EEG

## Abstract

As one of the most common neurological disorders, epilepsy causes great physical and psychological damage to the patients. The long-term recurrent and unprovoked seizures make the prediction necessary. In this paper, a novel approach for epileptic seizure prediction based on successive variational mode decomposition (SVMD) and transformers is proposed. SVMD is extended to multidimensional form for time-frequency analysis of multi-channel signals. It could adaptively extract common band-limited intrinsic modes among all channels on different time scales by solving a variational optimization problem. In the proposed seizure prediction method, data are first decomposed into multiple modes on different time scales by multivariate SVMD, and then, irrelevant modes are removed for preprocessing. Finally, power spectrum of denoised data is input to a pre-trained bidirectional encoder representations from transformers (BERTs) for prediction. The BERT could identify the mode information related to epileptic seizures in time-frequency domain. It shows fair prediction performance on an intracranial EEG dataset with the average sensitivity of 0.86 and FPR of 0.18/h.

## Introduction

Epilepsy is one of the most common brain diseases that affect people of all ages. The long-term recurrent and unprovoked seizures could cause great damage to physical and mental health of patients (Schulze-Bonhage and Kühn, 2008). An incoming seizure may be inhibited by some interventions such as medication and electrical or magnetic stimulation of the brain, if it is predicted in advance (Elger, 2001). Therefore, accurate prediction of epileptic seizure could not only significantly improve the quality of life for patients, but also provide a basis for the development of more effective methods of prevention and treatment of epilepsy. There are four phases of brain activity for patients: interictal phase (between seizures), preictal phase (prior to seizure), ictal phase (seizure), and postictal phase (after seizure). If the preictal state could be identified from other states, an imminent seizure will be predicted. The primary challenge in seizure prediction is the classification of preictal and interictal states (baseline). Electroencephalogram (EEG) is a method commonly used to diagnose epilepsy and evaluate its therapeutic effect (Fisher et al., 2005). In the recent years, an increasing number of literature demonstrates that there is a pattern in preictal EEG (Usman et al., 2019), and prediction of epileptic seizure by EEG is feasible.

Recently, the methods of seizure prediction have focused on time-frequency analysis, non-linear dynamics, and deep learning network. Common time-frequency analysis methods such as wavelet transform and empirical mode decomposition (EMD) have been applied to obtain EEG modes on different scales for seizure detection and prediction (Zahra et al., 2017; Zhang et al., 2018; Hassan et al., 2020; Savadkoohi et al., 2020). However, wavelet transform is not adaptive and the problems of EMD on low robustness and limited mathematical interpretation need to be improved (Dragomiretskiy and Zosso, 2013). Recently proposed variational mode decomposition (VMD) could separate the non-stationary signal into intrinsic modes with narrow band as well as EMD, but the advantages in complete mathematical theory framework and greater robustness (Dragomiretskiy and Zosso, 2013; Lahmiri, 2015) make it applied increasingly in various fields (Upadhyay and Pachori, 2015; Xue et al., 2016; Zhang et al., 2017; Li et al., 2018; Taran and Bajaj, 2018; Wang et al., 2019; Dora and Biswal, 2020; Guo et al., 2020), including epileptic seizure classification (Rout and Biswal, 2020; Peng et al., 2021). In addition to some statistical features in time domain and power spectral estimation in frequency domain, some non-linear dynamical parameters such as fractal dimension (Aarabi and He, 2017), largest Lyapunov exponent (Fei et al., 2017), fuzzy distribution entropy (Zhang et al., 2018), and Hjorth parameters (Teixeira et al., 2014) were also selected as features. Because it was difficult to describe preictal state with just a few features, many tedious feature engineering techniques were involved in the previous studies. However, some features were a lack of reproducibility and reliability (Mormann et al., 2005, 2007; Assi et al., 2017b). Recently, deep learning networks, including convolutional neural networks (CNN) and long short-term memory (LSTM) networks, have attracted most interest in seizure prediction, as their classification performance of preictal state and interictal state is superior to traditional machine learning techniques (Tsiouris et al., 2018; Usman et al., 2019). The latest bidirectional encoder representations from transformer (BERT) (Lee and Toutanova, 2018) is a very attractive deep learning network, which has made a great progress in the field of natural language processing (NLP). It has demonstrated superior performance over LSTM on many NLP tasks. Its application potential in other time series analysis is worth further exploring.

In this paper, a multidimensional extension of SVMD is proposed to adaptively extract common intrinsic modes among all channels on different time scales. After decomposed by multivariate SVMD, task-independent modes of the data could be removed for preprocessing or denoising. Then, the power spectrum of denoised iEEG data is input to a pre-trained BERT model for seizure prediction. The proposed seizure prediction method works well on two iEEG datasets.

The work is organized as follows. In Materials and methods, we introduce the information of database used in this paper and the proposed scheme, respectively. In addition, method of performance evaluation and seizure prediction are shown in this section. In Results, we present the experiments' results. In Discussion, we discuss the preprocessing method of SVMD and different seizure prediction methods used on the iEEG dataset. Finally, we conclude this paper in Conclusion.

## Materials and methods

### EEG dataset

The first dataset was obtained from Kaggle American Epilepsy Society Seizure Prediction Challenge (https://www.kaggle.com/competitions/seizure-prediction/). It is comprised of long-term intracranial EEG (iEEG) recordings from five dogs and two patients. Another dataset used in this study is comprised of continuous iEEG recordings from three dogs (Dog_6, Dog_7, and Dog_8), which could be obtained from NIH-sponsored International Epilepsy Electrophysiology portal (https://www.ieeg.org). The Canine iEEG data were sampled from 16 or 15 electrodes at 400 Hz. iEEG data of two patients were sampled at 5,000 Hz and recorded with 15 (Patient_1) and 24 (Patient_2) implanted electrodes, respectively. The type of seizures is focal epilepsy. More details were described in reference (Brinkmann et al., 2016). In this dataset, 1 h before seizure with a 5-min horizon (i.e., 66–5 min before seizure onset) was chosen as preictal phase (Brinkmann et al., 2016; Assi et al., 2017a; Gagliano et al., 2019; Nejedly et al., 2019; Yu et al., 2021). Each consecutive interictal sequence lasted for 1 h, which were randomly chosen from iEEG recordings more than 1 week (dogs) and 4 h (patients) before or after any seizure. The iEEG portal dataset is comprised of continuous iEEG recordings, which are all labeled. The Kaggle dataset consists of training data and testing data. Each labeled iEEG sequence of training data lasts for 1 h, and unlabeled testing data are 10-min iEEG segment (the contest website does not have labels for test data, and the score could only be obtained by uploading the predicted results of all test data to the website). The description of the data used in this work is shown in Table 1.

**Table 1:** Description of the Kaggle dataset.

**Participant**	**No. of**	**No. of**	**Interictal**	**No. of**
	**channels**	**seizures**	**hours**	**testing segments**
Dog_1	16	4	80	502
Dog_2	16	7	83	1,000
Dog_3	16	12	240	907
Dog_4	16	16	134	990
Dog_5	15	5	75	191
Dog_6	16	41	998	-
Dog_7	16	38	936	-
Dog_8	16	15	286	-
Patient_1	15	2	8.3	195
Patient_2	24	3	7	150

### Preprocessing methods

The multidimensional extension of successive variational mode decomposition (SVMD) is proposed for time-frequency analysis of non-stationary multi-channel signals in this section. Multivariate SVMD is used to remove irrelevant modes for denoising in the presented seizure prediction method.

Successive variational mode decomposition is established under the similar theoretical framework as VMD, which requires each extracted mode to be compact around its center frequency and original data to be reconstructed by all modes. However, different from VMD, SVMD could successively decompose each intrinsic mode from a signal without specifying the number of modes in advance. Therefore, there is no complex multi-parameter optimization problem for SVMD. Details of the algorithm could be found in the reference (Nazari and Sakhaei, 2020). As there is also a lot of demand for analyzing multi-channel signals in real-world applications, a simple multivariate extension of SVMD is presented.

Multidimensional SVMD aimed to adaptively extract common intrinsic modes **u**_*i*_(*t*) with limited bandwidth from multivariate signal ***f***(*t*) containing *C* channels, i.e., ***f***(*t*)**=**[*f*_1_(*t*), *f*_2_(*t*), …, *f*_*c*_(*t*)].


(1) $$\begin{array}{l} f\left(t\right)=\sum_{i=1}^{L}{\text{u}}_{i}\left(t\right) \end{array}$$

where **u**_*i*_(*t*) = [*u*_*i*1_(*t*), *u*_*i*2_(*t*), …, *u*_*ic*_(*t*)], *C* is the number of channels and *L* is the number of common modes decomposed by multivariate SVMD.

It is noteworthy that intrinsic modes on the ***l***th scale *u*_*l*_(*t*) are set to the same central frequency ω_*l*_ in our model for the purpose of getting common modes of *C* channels on the same time scale. According to the definition of intrinsic mode function, *u*_*i*_(*t*) should be limited bandwidth signals, which is the central assumption for mode separation in SVMD. Therefore, the average bandwidth of all modes on the ***l***th time scale should be minimized. Equivalently, the total bandwidth of *C* modes forms cost function *L*_1_ in multivariate SVMD optimization problem and is given by


(2) $$\begin{array}{l} L_{1}=\sum_{k=1}^{C}\|\partial_{t}\left[\left(\delta \left(t\right)+\frac{j}{\pi t}\right)\;{*\;u}_{lk}\left(t\right)\right]e^{-j\omega_{l}t}{\|}_{2}^{2} \end{array}$$

To obtain the complete modes on the ***l***th scale and avoid mode mixing with other scales, neither the previously extracted *l* − 1 modes nor undecomposed part *f*_*uk*_(*t*) of the ***k****th* channel (*k* = 1, 2, …, *C*) should contain any information of the ***l***th mode. Meanwhile, there should be no spectral overlap between the ***l***th mode and previously decomposed *l* − 1 modes. Accordingly, criteria *L*_2_, namely, the total frequency response of residual signals (${\left\{u_{ik}\left(t\right)\right\}}_{i=1}^{l-1}\;$and *f*_*uk*_(*t*)) of all channels after passing through the filter ${\hat{\beta }}_{l}\left(\omega \right)$(frequency response of the *lth* filter), should be minimized. Furthermore, for the ***k****th* channel, the total energy of filtered *u*_*lk*_(*t*) by each filter ${\hat{\beta }}_{i}\left(\omega \right)$ (*i* = 1, 2, …*l* − 1) requires as less as possible. This constraint is shown in the cost function *L*_3_.


(3) $$\begin{array}{l} L_{2}=\sum_{k=1}^{C}\|\beta_{l}\left(t\right)\;*\;\left(f_{uk}\left(t\right)+\sum_{i=1}^{l-1}u_{ik}\left(t\right)\right){\|}_{2}^{2} \end{array}$$

(4) $$\begin{array}{l} L_{3}=\sum_{k=1}^{C}\sum_{i=1}^{l-1}\|\beta_{i}\left(t\right)\;*\;u_{lk}\left(t\right){\|}_{2}^{2} \end{array}$$

(5) $$\begin{array}{l} {\hat{\beta }}_{i}\left(\omega \right)=\frac{1}{\alpha {\left(\omega -\omega_{i}\right)}^{2}}\;i=1,2,\ldots ,\;L \end{array}$$

The constrained variational optimization problem for multivariate SVMD is represented as follows:


(6) $$\begin{array}{l} \;\begin{array}{c} \underset{u_{lk},\omega_{l},f_{uk}(t)}{\min}\alpha L_{1}+L_{2}+L_{3}\;\\ \;s.t.\;u_{lk}\left(t\right)+f_{uk}\left(t\right)+\sum_{i=1}^{l-1}u_{ik}\left(t\right)=f_{k}\left(t\right),\;\\ \;k=1,2,\ldots ,C \end{array}\;\} \end{array}$$

The augmented Lagrange function shown in (7) is used to transform this problem into unconstrained optimization problem, which could be solved iteratively by ADMM approach (Bertsekas, 1982)


(7) $$\begin{array}{l} \;\begin{array}{c} L\left(u_{lk},\omega_{l},\lambda_{k}\right)=\alpha L_{1}+L_{2}+L_{3}\;\\ +\sum_{k=1}^{C}\|f_{k}\left(t\right)-\left(u_{lk}\left(t\right)+f_{uk}\left(t\right)+\sum_{i=1}^{l-1}u_{ik}\left(t\right)\right){\|}_{2}^{2}\;\\ +\sum_{k=1}^{C}\langle \lambda_{k}\left(t\right),f_{k}\left(t\right)-\left(u_{lk}\left(t\right)+f_{uk}\left(t\right)+\sum_{i=1}^{l-1}u_{ik}\left(t\right)\right)\rangle \end{array}\} \end{array}$$

The first subproblem is focused on updating the modes *u*_*lk*_ iteratively by channel. The **(*n* + 1)***th* iteration of the ***k***th channel could be rewritten as the following equivalent problem, which is actually reduced to a univariate mode update problem in original SVMD.


(8) $$\begin{array}{l} \;{\hat{u}}_{lk}^{n+1}(t)=\underset{\text{lk}}{\arg}\min\{\alpha {\left\|\partial_{t}\left[\left(\delta (t)+\frac{j}{\pi t}\right)\;*\;u_{lk}(t)\right]e^{-j\omega_{l}t}\right\|}_{2}^{2}\\ +{\left\|\beta_{l}(t)\;*\;\left(f_{uk}(t)+\sum_{i=1}^{l-1}u_{ik}(t)\right)\right\|}_{2}^{2}+\sum_{i=1}^{l-1}{\left\|\beta_{i}(t)\;*\;u_{lk}(t)\;\right\|}_{2}^{2}\\ \;+\sum_{k=1}^{C}{\left\|f_{k}(t)-\left(u_{lk}(t)+f_{uk}(t)+\sum_{i=1}^{l-1}u_{ik}(t)\right)\right\|}_{2}^{2}\\ \;+\sum_{k=1}^{C}\langle \lambda_{k}(t),f_{k}(t)-\left(u_{lk}(t)+f_{uk}(t)+\sum_{i=1}^{l-1}u_{ik}(t)\right)\rangle \} \end{array}$$

Therefore, as same as SVMD, it could be solved in spectral domain based on the Parseval's equality. *u*_*lk*_ is updated by (9). Details could be found in reference (Nazari and Sakhaei, 2020).


(9) $$\begin{array}{l} {û}_{lk}^{n+1}\left(\omega \right)=\frac{{\hat{f}}_{k}\left(\omega \right)+\alpha^{2}{\left(\omega -\omega_{l}^{n}\right)}^{4}{û}_{lk}^{n}\left(\omega \right)+\frac{{\hat{\lambda }}_{k}}{2}}{\left[1+\alpha^{2}{\left(\omega -\omega_{l}^{n}\right)}^{4}\right]\left[1+2\alpha {\left(\omega -\omega_{l}^{n}\right)}^{2}+\sum_{i=1}^{l-1}\frac{1}{\alpha^{2}{\left(\omega -\omega_{i}\right)}^{4}}\right]} \end{array}$$

The second subproblem is related to updating the center frequency ω_*l*_. The **(*n* + 1)***th* iteration of each channel is the minimization problem shown in (10), which could be solved with the method and equation applied in SVMD. According to the principle of linear superposition, ω_*l*_ could be updated by Equation (11).


(10) $$\begin{array}{l} \omega_{l}^{n+1}={arg}_{\omega_{l}}\min\left\{\alpha L_{1}+L_{2}\right\} \end{array}$$

(11) $$\begin{array}{l} \omega_{l}^{n+1}=\frac{\sum_{k=1}^{C}\int_{0}^{\infty }{\omega |{û}_{lk}^{n+1}\left(\omega \right)|}^{2}d\omega }{\sum_{k=1}^{C}\int_{0}^{\infty }|{û}_{lk}^{n+1}\left(\omega \right){|}^{2}d\omega } \end{array}$$

The updating equation of Lagrange multiplier λ is the same as SVMD, as long as replace û_*i*_ by û_*ik*_.

The result of decomposition is affected by the penalty factor α, which determines the bandwidth of intrinsic modes (Dragomiretskiy and Zosso, 2013; Nazari and Sakhaei, 2020). Furthermore, the optimal α differs obviously when decomposing different types of signals. Consequently, a heuristic method similar to SVMD is introduced to obviate optimization of α. In the iteration of extracting modes of the ***l****th* scale, α is set to grow exponentially from a small value α_min_ to a maximum allowable value α_max_, which is actually a process of finding the strongest modes in the residual signals from coarse to fine tuning.

The algorithm terminates search until total energy of all the ***l***th modes is less than the given threshold ε_2_; namely, the modes extracted could be regarded as noise. Finally, all the obtained modes are sorted by their center frequency from low to high. The complete algorithm for multivariate SVMD is described in Table 2.

**Table 2:** The complete algorithm of multivariate SVMD.

**The algorithm of SVMD**
Initialize: *l*←0, $\varepsilon_{1}\leftarrow 10^{-6}$, $\varepsilon_{2}\leftarrow 10^{-3}$ **repeat** *l*←*l* + 1 Set ${û}_{Lk}^{1}\left(\omega \right)$, ${\hat{\lambda }}_{k}^{1}$, $\omega_{L}^{1}$, *n*←0, *m*←0, α_1_←α_min_ **repeat** *m*←*m* + 1 **repeat** *n*←*n* + 1 **for** *k* = 1 : *C* **do** Update *u*_*lk*_ for all *ω ≥* 0: ${û}_{lk}^{n\;+\;1}\left(\omega \right)=\frac{{\hat{f}}_{k}\left(\omega \right)\;+\;\alpha^{2}{\left(\omega -\omega_{l}^{n}\right)}^{4}{û}_{lk}^{n}\left(\omega \right)\;+\;\frac{{\hat{\lambda }}_{k}}{2}}{\left[1\;+\;\alpha^{2}{\left(\omega -\omega_{l}^{n}\right)}^{4}\right]\left[1\;+\;2\alpha {\left(\omega -\omega_{l}^{n}\right)}^{2}\;+\;\sum_{i=1}^{l-1}\frac{1}{\alpha^{2}{\left(\omega -\omega_{i}\right)}^{4}}\right]}$ **end for** **for** *k* = 1 : *C* **do** Update ω_*l*_ $\omega_{l}^{n+1}=\frac{\sum_{k=1}^{C}\int_{0}^{\infty }{\omega |{û}_{lk}^{n+1}\left(\omega \right)|}^{2}d\omega }{\sum_{k=1}^{C}\int_{0}^{\infty }|{û}_{lk}^{n+1}\left(\omega \right){|}^{2}d\omega }$ **end for** **for** *k* = 1 : *C* **do** Dual Ascent for all *ω ≥* 0: ${\hat{\lambda }}_{k}^{n+1}={\hat{\lambda }}_{k}^{n}+\tau [{\hat{f}}_{k}\left(\omega \right)-({û}_{lk}^{n+1}\left(\omega \right)$ $+\frac{\alpha^{2}{\left(\omega -\omega_{l}^{n}\right)}^{4}\left({\hat{f}}_{k}\left(\omega \right)-{û}_{lk}^{n+1}\left(\omega \right)-\sum_{i=1}^{l-1}{û}_{ik}\left(\omega \right)+\frac{{\hat{\lambda }}_{k}}{2}\right)-\sum_{i=1}^{l-1}{û}_{ik}\left(\omega \right)}{1+\alpha^{2}{\left(\omega -\omega_{l}^{n+1}\right)}^{4}}+\overset{l-1}{\underset{i=1}{\sum }}{û}_{ik}\left(\omega \right))]$ **end for** **Until** convergence: $\frac{\|{û}_{lk}^{n+1}\left(\omega \right)-{û}_{lk}^{n}\left(\omega \right){\|}_{2}^{2}}{\|{û}_{lk}^{n}\left(\omega \right){\|}_{2}^{2}}<\varepsilon_{1},\;k\;=1,\;2,\;\ldots ,\;C$ Set ${\hat{\lambda }}_{k}^{1}$, ${û}_{lk}^{1}\left(\omega \right)\leftarrow {û}_{lk}^{n+1}\left(\omega \right)$, $\omega_{l}^{1}\leftarrow \omega_{l}^{n+1}$, $\alpha_{m}\leftarrow \alpha_{\min}+e^{m}$, *n*←0 **Until** α_*m*_ ≤ α_max_ **Until** $\sum_{k=1}^{C}\left[\frac{1}{T}\|u_{lk}\left(t\right){\|}_{2}^{2}\right]<\varepsilon_{2}$

### Classification and evaluation

The human iEEG data were down-sampled to 500 Hz to be comparable to canine iEEG. To reduce computational burden of SVMD, both preictal and interictal iEEG data were first divided into 2-s clips without overlap. Then, all iEEG clips were decomposed by multivariate SVMD. Irrelevant modes of raw iEEG data were removed and the remaining ones were added up for reconstruction. Subsequently, the reconstructed data were concatenated into a new time series in chronological order. The denoised iEEG data were split into 30-s-long samples with 28-s overlap. To use modal information in time-frequency domain for prediction, power spectrum was extracted by the short-time Fourier transform (STFT). Each iEEG sample was segmented by a 1-s time window with 75% overlap to compute the power spectrum by the function *spectrum* in MATLAB. Only the power spectrum from 0 to 140 Hz is selected in this study, and the average of the power per 2 Hz is calculated as the final spectrum. The power spectrum of iEEG samples was input to a deep learning network based on BERT for seizure prediction. To compare the performance of preprocessing, the power spectrum of raw iEEG was also input to BERT for classification.

#### BERT model architecture

**Figure 1:**
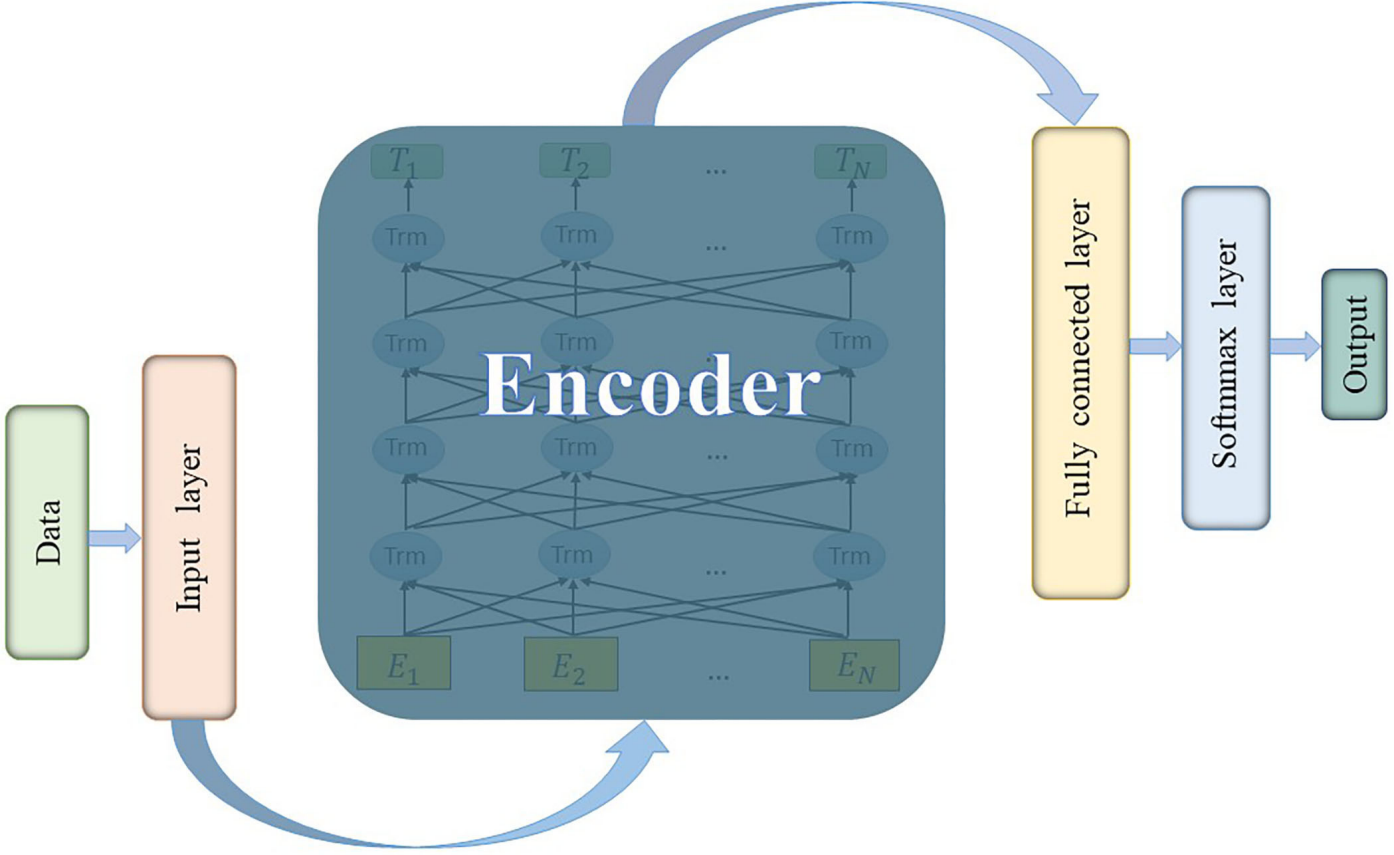
The architecture of BERT model.

The classic BERT's model architecture is based on a multi-layer bidirectional transformer encoder (Vaswani et al., 2017) and it uses bidirectional self-attention mechanism. After being pre-trained with two unsupervised tasks, all parameters of BERT could be fine-tuned using labeled data from the downstream tasks. The code and pre-trained models are available at https://github.com/matlab-deep-learning/transformer-models. In this study, the classification of preictal and interictal iEEG could be considered as a downstream task to finetune a pre-trained BERT model with an additional output layer. Our model architecture consists of input layer, encoder layer (transformer blocks), fully connected layer, and Softmax classification layer, as shown in Figure 1.

It is worth noting that BERT is originally designed to solve NLP tasks, and the input representation is a token sequence transformed from a sentence (Wu et al., 2016). However, the input data are essentially a digital time series, which is unnecessary to convert to tokens and then use word embedding in the input layer. Therefore, a more suitable embedding method for digital sequence needs to be designed. The input data of all channels are concatenated and weighted as a kind of data embedding [refer to Equations (12) and (13)], which could be considered as a kind of data fusion.


(12) $$\begin{array}{l} x_{j}=\left[\begin{array}{c} \begin{array}{c} {\tilde{x}}_{1j}\\ {\tilde{x}}_{2j}\\ \vdots \end{array}\\ {\tilde{x}}_{Ncj} \end{array}\right],\;X=\left[x_{1},{x_{2},\ldots ,x}_{N}\right],\;j\;=\;1,\;2,\;\ldots ,\;N \end{array}$$

(13) $$\begin{array}{l} E_{d}\;=\;X⊙W \end{array}$$

where ${\tilde{x}}_{ij}$ is power spectrum of the ***i****th* channel in the ***j****th* time window (each 1-s time window is set as a time step, and *N* is the number of time steps), and all channels are cascaded to construct a (*N*_*c*_ × *N*_*p*_) × 1 vector (*N*_*c*_ is the number of channels, and *N*_*p*_ is the number of spectrum frequencies). The Hadamard product of the power spectrum *X* and weight matrix *W* is the data embedding *E*_*d*_.

In the input layer, *X* is converted to a matrix *E* by summing the position embedding *E*_*p*_ (the embedding method is the same as BERT) and data embedding *E*_*d*_. Dynamic coding is applied and all weights are automatically learned by training. Weights are first initialized as random numbers that obey normal distribution. After embedding and normalization, the (*N*_*c*_ × *N*_*p*_) × *N* (i.e., number of features × number of time steps) matrix is input to the encoder layer.

In the encoder layer, the number of layers (i.e., transformer blocks) is 12 and the hidden size is 768. The number of self-attention heads is 12. Batch size is set to 32 and the number of epochs in training loop is 10. The BERT model is built with MATLAB R2022a.

#### Evaluation

To test the predictive ability of this approach for unknown seizures, limited seizures were used for training, whereas the remaining ones were for testing. All the data of iEEG portal dataset and labeled training data in Kaggle dataset could be used. Because there were relatively few seizures for each subject in the training data, a leave-one-out cross-validation method was applied. Namely, *M*-1 seizures were used for training and one for validation if there were *M* seizures for a subject. The amount of interictal iEEG is much larger than preictal iEEG. Therefore, to avoid the problem of class imbalance, a number of preictal and interictal iEEG sequences were the same in the training set. Each interictal iEEG sequence was randomly selected from the dataset. All remaining interictal sequences were used for validation. We run ten trials and train 10 models for each subject (refer to Lian et al., 2020). The average performance was considered as final prediction performance when using training data. We could also use unlabeled testing data of Kaggle dataset to test the prediction method. Similarly, we trained multiple models to avoid the problem of class imbalance. For each subject, all preictal iEEG and the same amount of randomly selected interictal iEEG were used to train the model, and we run 10 trials. A testing segment in Kaggle dataset would be predicted as preictal iEEG, if more than 6 models identified it as preictal. No labels are given for the testing data in Kaggle dataset, but the score (an index related to classification accuracy that used by the organizer) could be calculated on the competition website. Therefore, the score we achieved on testing data is a key indicator of predictor performance.

To improve the reliability of the prediction, a prediction window of 10 min was applied. According to experiential knowledge [refer to (Truong et al., 2018; Wei et al., 2019)], if more than 60% of EEG samples during 10-min continuous recordings are identified as preictal, the warning alarm would be raised. To evaluate the performance of the prediction method, there are four commonly used measures including sensitivity, false prediction rate (FPR), seizure occurrence period (SOP), and seizure prediction horizon (SPH). Sensitivity is the number of correctly predicted seizures divided by the total number of seizures. FPR is defined as the number of false alarms per hour. SPH is a predefined interval between the first alarm and the incoming seizure, which is also a period reserved for patients to take intervention measures. SOP is the period during which a seizure is expected to occur (Maiwald et al., 2004). Therefore, for a correct prediction, seizure would not occur during the SPH and must occur within the SOP. There are no common criteria for the length of SOP and SPH, but the SPH should be long enough for intervention and the SOP should not be too long in case of patient's anxiety. Based on prior knowledge of other studies, we use the SPH of 30 min and the SOP of 20 min here.

To evaluate the statistical significance of the seizure prediction performance, a random predictor is used for comparison. For a given FPR, the probability to raise an alarm during the SOP can be approximated as follows: (Schelter et al., 2006).


(14) $$\begin{array}{l} P\approx 1-e^{-FPR\cdot SOP} \end{array}$$

Therefore, the probability of predicting at least *m* of *M* independent seizures by chance is given by


(15) $$\begin{array}{l} p=\sum_{i\geq m}\left(\begin{array}{c} M\\ i \end{array}\right)P^{i}{\left(1-P\right)}^{M-i} \end{array}$$

For each patient, p is calculated using the FPR and the number of correctly predicted seizures *m*. If *p* is < 0.05, the prediction method is considered significantly better than a random predictor at a significance level of 0.05.

## Results

### Preprocessing results

**Figure 2:**
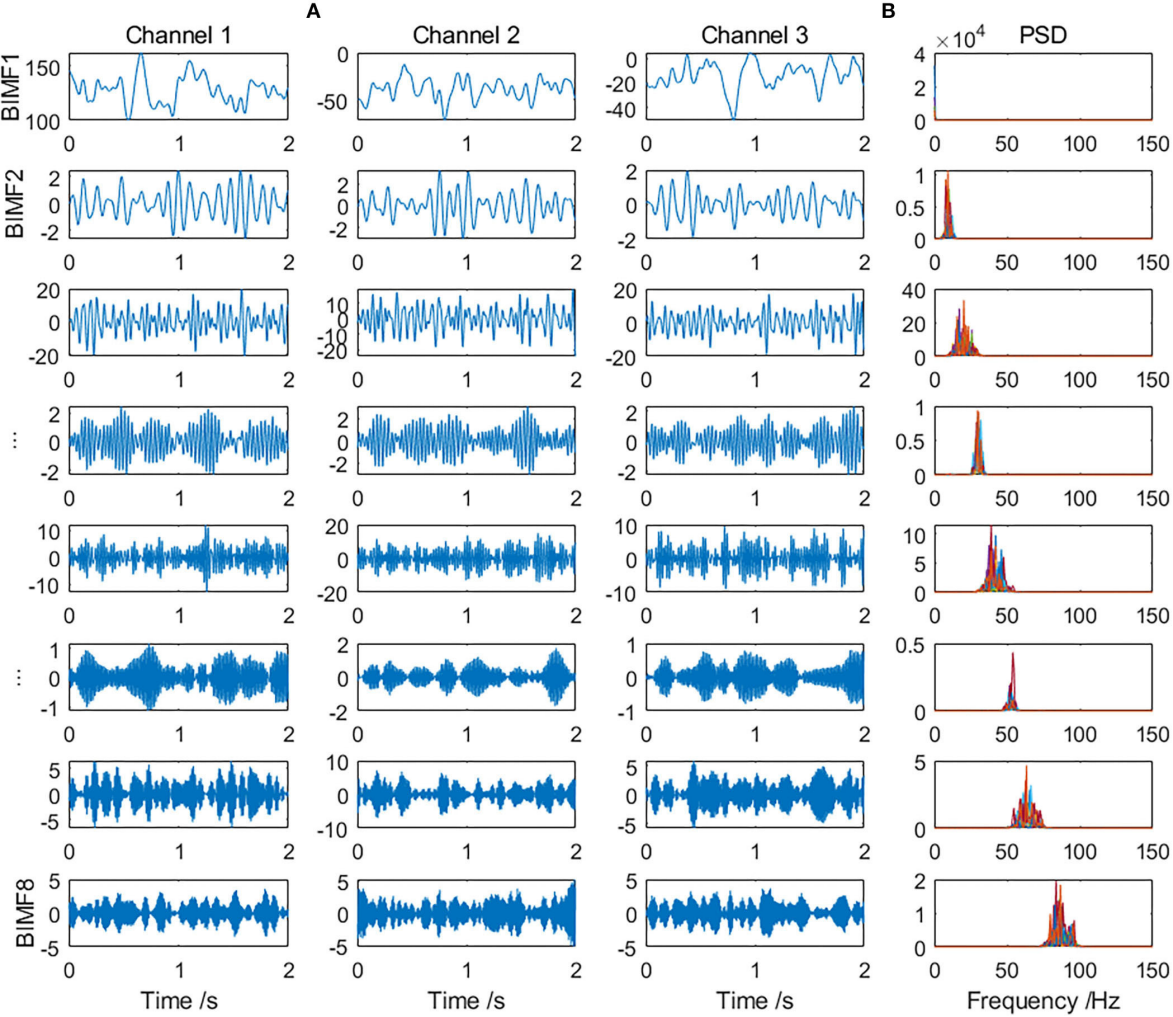
**(A)** The eight scales of modes extracted from a randomly selected preictal iEEG sample by multivariate SVMD (only the first 3 channels are displayed) and **(B)** PSD of all 15 channels on each scale.

Multivariate SVMD was applied for preprocessing. The range of parameter α in SVMD was set to [200, 800] for canine iEEG and [200, 2000] for patient iEEG. The eight scales of common intrinsic modes extracted from a randomly selected 2-s preictal iEEG of Dog_5 are shown in Figure 2A (only the first 3 channels are displayed in the figure due to space limitations). Corresponding power spectrum density (PSD) of all 15 channels on each scale is indicated in Figure 2B. The frequency bands of modes on the same scale were similar, which illustrated the mode-alignment ability of multivariate SVMD across multiple channels. The modes which could obtain the highest classification accuracy were considered as effective modes and the others were irrelevant. Irrelevant modes were removed and the remaining modes were added up for reconstruction. It could be considered a kind of denoising.

### Prediction results

The power spectrum of reconstructed data was input to BERT for deep learning and classification. It is shown in Table 3 that this prediction algorithm achieves mean sensitivity of 0.86 and the average FPR of 0.18/h. The *p*-value indicated that the prediction method was significantly superior to a random predictor for all subjects. The mean score of our method on testing data of Kaggle dataset was 0.84125, which was about 0.03 below the competition leader of 0.87154. The power spectrum of raw iEEG was also input to BERT for classification, to compare the preprocessing algorithms. The mean score on testing data was 0.69153, which is, however, much lower than the proposed method.

**Table 3:** The performance of the proposed method on 10 subjects.

**Participant**	**No. of seizures**	**Center frequency of removed modes (Hz)**	**Sensitivity**	**FPR (/h)**	**p**
Dog_1	4	8 < ω < 15	0.65	0.25	0.0019
Dog_2	7	12 < ω < 20	0.87	0.06	< 0.001
Dog_3	12	12 < ω < 20	0.92	0.23	< 0.001
Dog_4	16	ω < 60	0.94	0.07	< 0.001
Dog_5	5	ω < 55	0.90	0.16	< 0.001
Dog_6	41	ω < 30	0.90	0.15	< 0.001
Dog_7	38	ω < 30	0.86	0.18	< 0.001
Dog_8	15	ω < 20	0.88	0.09	< 0.001
Patient_1	2	ω < 55	1	0.36	0.0128
Patient_2	3	ω < 30	0.67	0.25	0.0182
Mean			0.86	0.18	

## Discussion

**Figure 3:**
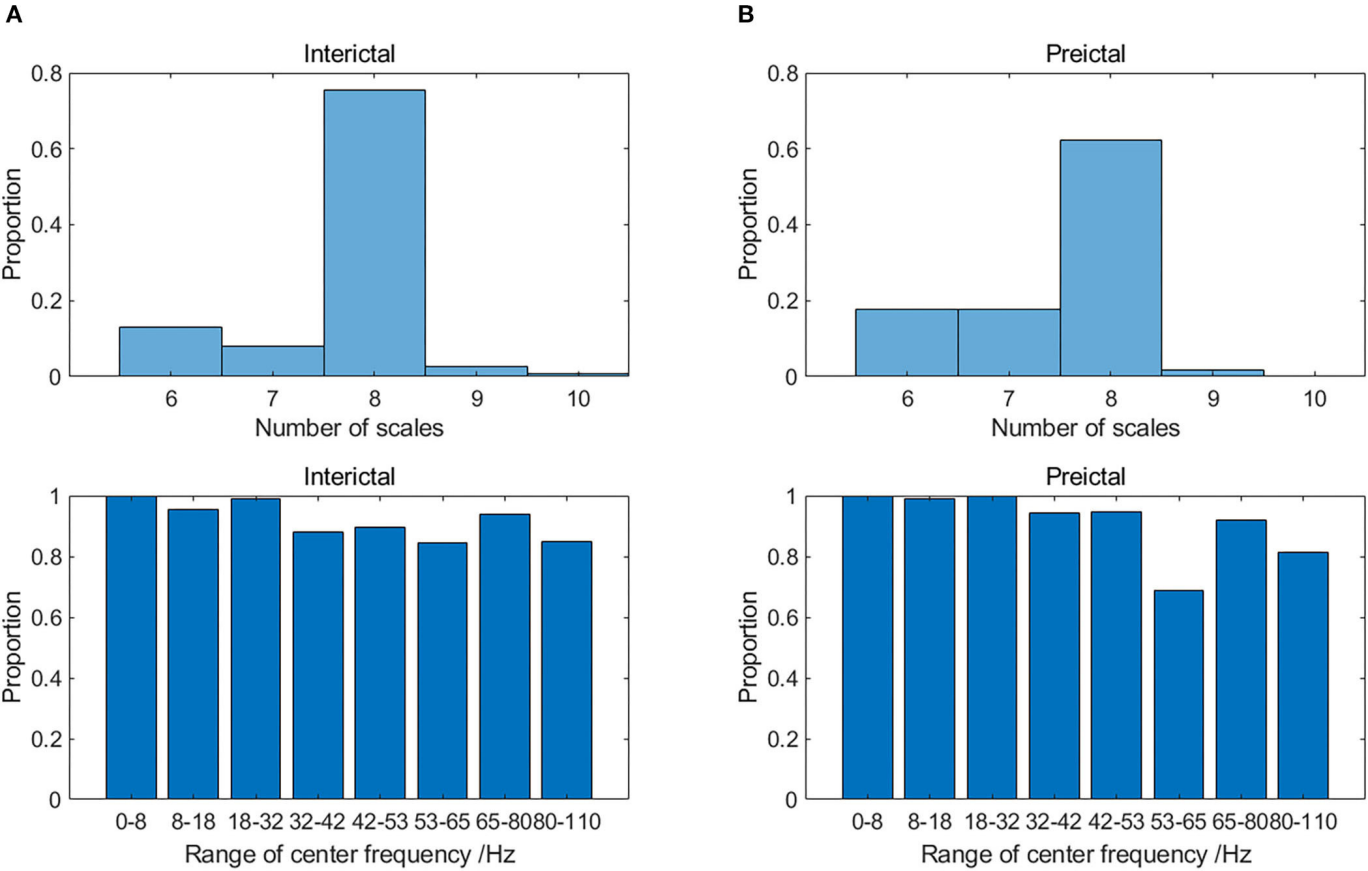
The distribution of the number of time scales (upper) and range of center frequency on 8 dominant scales (lower) in **(A)** interictal and **(B)** preictal states for Dog_5.

Multivariate SVMD inherits the advantages of SVMD including less parameters, resistance to mode mixing and adaptability. Meanwhile, it could be seen from Figure 3 that the frequency bands of modes on the same scale were similar, which illustrated the mode-alignment ability of multivariate SVMD across multiple channels. Furthermore, modes in different scales were in distinctive frequency bands, which demonstrated that SVMD might have filter bank property, which is not the focus of this study, but could be further proofed in the future.

The number of the intrinsic modes extracted by multivariate SVMD for some samples was not consistent, because of wideband iEEG signals with the effect of ocular artifacts, electromyogram, and other background noise. Take the data of Dog_5 for example, the distribution of the number of time scales and center frequency on 8 dominant scales are displayed in Figure 3. Most of the interictal samples (75.6%) were decomposed into 8 scales of band-limited intrinsic mode function (BIMF), whereas there was less consistency for preictal samples on the number of modes. For both states, the center frequencies of 8 dominant time scales were in the range of [0, 8], [8, 18], [18, 32], [32, 42], [42, 53], [53, 65], [65, 80], and [80, 110] respectively. However, the proportion of samples containing certain time scales of modes (modes in high gamma band) is significantly reduced in the preictal state, as shown in Figure 3. The reason might be that some modes were interfered by the new modes generated by an impending epileptic seizure, which needs to be proved by exploiting more physiological evidence.

It can be seen from Table 3 that the difference of preictal and interictal modes shows specificity among all subjects. There is a certain consistency for Dog_1, Dog_2, and Dog_3, because all the irrelevant modes are in alpha and beta bands. However, modes that are associated with seizures are in gamma band for other subjects. Therefore, the seizure prediction method is patient-dependent due to the specificity of patients.

As summarized in the reference (Usman et al., 2019), support vector machine (SVM) was widely used in studies before 2019 with good predictive performance. LSTM was the most commonly used model among deep learning models to solve NLP problems and other time series pattern recognition before the emergence of BERT. Therefore, we compare the prediction ability of these two classifiers with BERT. SVM with Gaussian radial basis function (RBF) kernel is used by reference to the literature (Bandarabadi et al., 2015; Xiang et al., 2015; Sharif and Jafari, 2017). The LSTM network is consisted of a sequence input layer, a LSTM layer, a dropout layer, a fully connected layer using the “relu” activation function, and a classification layer using the “softmax” activation. The size of input layer is dependent on the number of power spectrum features. The dropout probability is 0.5. The number of memory units on the LSTM layer is set to 128 (Tsiouris et al., 2018). Although the mean sensitivity of SVM could reach 0.83, the score on testing data is only 0.65839. The prediction performance of both LSTM and BERT on testing data is much better than that of SVM, which may due to the stronger learning ability of the two deep learning models for temporal information. Moreover, BERT could achieve better prediction results than LSTM, as shown in Table 4. It illustrates that BERT shows better performance in epileptic seizure prediction than LSTM.

**Table 4:** The prediction performance of three classifiers (SVM, LSTM, and BERT).

	**Training data**		**Testing data**
	**Sensitivity**	**FPR**	**score**
SVM	0.83	0.24	0.65839
LSTM	0.84	0.21	0.77930
BERT	0.86	0.18	0.84125

The sensitivity and FPR in the table are the average of 10 subjects.

As is shown in Table 5, for the canine iEEG dataset, the sensitivity of this method is higher than that of other methods, and the FPR of 0.20 is relatively low. It represents the high prediction performance of this method. The mean score achieved on testing data was 0.84125 with preprocessed data, while only 0.69153 with raw iEEG data, which illustrates that SVMD could screen out valid modes for seizure prediction. Meanwhile, it proved again that the difference between preictal state and interictal state of brain exists in the power spectrum of iEEG in time-frequency domain. The self-attention learning mechanism of BERT could extract the information effectively. Although the result is comparable with the work of Assi et al. there were only three subjects and complex feature extraction, and feature selection and channel selection were used to predict seizures in that study. However, there are 10 subjects in the two datasets we used, and our method is relatively simple. Only the power spectrum of denoised iEEG by SVMD is used as features for prediction.

**Table 5:** Comparison of seizures prediction methods using iEEG dataset.

**Authors**	**No. of subjects**	**Features**	**Classifier**	**Sensitivity**	**FPR**
Assi et al. (2017a)	3	Spectral band power, Hjorth mobility and complexity, spectral edge frequency and power, and decorrelation time	SVM	0.85	-
Truong et al. (2018)	7	Short-time Fourier transform	CNN	0.75	0.21
Nejedly et al. (2019)	4	Raw iEEG and spectrogram images	CNN	0.79	-
Gagliano et al. (2019)	3	Higher-order spectral features	LSTM	0.78	-
Yu et al. (2021)	7	Autoregressive (AR) model coefficients and Laguerre–Volterra AR model coefficients	Sparse lasso logistic regression classifier	0.78	-
This work	10	Power spectrum of reconstructed data by multivariate SVMD	BERT	0.86	0.18

The mean score obtained by the first team is 0.87154 in the Kaggle competition. Although it is about 0.03 higher than our method, their result is based on the numerous features and elaborate feature selection. The features include energy in different frequency bands, correlation of energy between channels, square root of each feature, and so on (they only briefly introduced the features in the following websites: https://www.kaggle.com/competitions/seizure-prediction/discussion/11024). However, features are learned adaptively in our method. The score we achieved indicates that the proposed approach could be a candidate or auxiliary method for seizure prediction.

## Conclusion

In this paper, we proposed a seizure prediction method based on SVMD and BERT. The simple extension of SVMD could decompose multivariate data into its common inherent modes on different scales. The iEEG signals were preprocessed by removing irrelevant modes after decomposition by SVMD. The prediction score on Kaggle competition indicated that BERT could learn the difference of preictal and interictal state in time-frequency domain using the self-attention learning mechanism. Therefore, it could be a candidate method for seizure prediction.

## Data availability statement

The original contributions presented in the study are included in the article/supplementary material, further inquiries can be directed to the corresponding author.

## Author contributions

XW and TZ designed the study. XW downloaded and analyzed the data, performed experiments, and drafted the manuscript. LZ, LQ, and TZ revised the manuscript. All authors read and approved the final manuscript.

## Funding

This work was partly supported by the National Natural Science Foundation of China (Nos. 61976110, 62176112, and 11931008), the Natural Science Foundation of Shandong Province (No. ZR202102270451), and The Open Project of Liaocheng University Animal Husbandry Discipline (No. 319312101-01).

## Conflict of interest

The authors declare that the research was conducted in the absence of any commercial or financial relationships that could be construed as a potential conflict of interest.

## Publisher's note

All claims expressed in this article are solely those of the authors and do not necessarily represent those of their affiliated organizations, or those of the publisher, the editors and the reviewers. Any product that may be evaluated in this article, or claim that may be made by its manufacturer, is not guaranteed or endorsed by the publisher.
